# The impact of CGRP monoclonal antibodies on cytokine expression in chronic migraine: a cohort study

**DOI:** 10.1007/s00415-025-13400-w

**Published:** 2025-09-24

**Authors:** Jason C. Ray, Stuart McDonald, Marian Todaro, Josephine Baker, Wei Zhen Yeh, Elspeth J. Hutton, Manjit Matharu, Helmut Butzkueven

**Affiliations:** 1 Department of Neurosciences, School of Translational Medicine, Monash University, Melbourne, Australia; 2 Department of Neurology, Alfred Health, Melbourne, Australia; 3 Department of Neurology, Austin Health, Melbourne, Australia; 4 Headache and Facial Pain Group, University College London (UCL) Queen Square Institute of Neurology and The National Hospital for Neurology and Neurosurgery, Queen Square, London, WC1N 3BG UK

**Keywords:** Migraine, Headache disorders, CGRP, Immune, Cytokine

## Abstract

**Background:**

Calcitonin gene-related peptide monoclonal antibodies (CGRP mAbs) are an effective preventative therapy for migraine; however, there have been rare reports of possible inflammatory complications. The primary objective of this study was to examine the impact of CGRP mAbs on immune system activation by evaluating the plasma cytokine profile of a cohort of patients pre- and post-CGRP mAb.

**Methodology:**

A prospective cohort study was undertaken at a tertiary headache service. Following informed consent and screening, the plasma cytokine profile of participants was determined using a Simoa CorPlex human cytokine 10-plex with ten targets: interferon gamma, interleukin-1β, IL-4, IL-5, IL-6, IL-8, IL-10, IL-12p70, IL-22, and TNF⍺ prior to initiation of CGRP mAb and following 3 months of therapy. A comparator group of healthy controls at a single time point was also included.

**Results:**

A total of 22 patients with chronic migraine and 10 healthy controls were included in the study. Administration of CGRP mAb was not associated with a significant change in cytokine expression (Wilk’s lambda 0.528, *p* = 0.448). On post-hoc analysis, there was a significant reduction in IL-5 levels (*z* = − 2.321, *p* = 0.020) following CGRP mAb therapy.

**Conclusion:**

In this study of patients with chronic migraine, we found no evidence that treatment with CGRP mABs is associated with a significant alteration in plasma cytokine levels or shift to a Th1 phenotype.

**Supplementary Information:**

The online version contains supplementary material available at 10.1007/s00415-025-13400-w.

## Introduction

The calcitonin gene-related peptide receptor is expressed widely throughout the body and has multiple biological functions [1]. While the inhibition of CGRP or its canonical receptor has been demonstrated to be an effective therapy in migraine, ongoing research is required to assess for possible off-target effects of calcitonin gene-related peptide monoclonal antibodies (CGRP mAbs) to ensure their continued safe use [1].

In 2021, we published a report of a series of patients who developed new inflammatory complications following treatment with a CGRP monoclonal antibody [2]. The case series reported disparate inflammatory complications, including several severe flares of psoriasis/psoriatic arthritis, autoimmune hepatitis, and granulomatosis with polyangiitis. These cases all were temporally related to the initiation of a CGRP mAb, led to cessation, and subsequently improved post cessation, highlighting the pre-clinical evidence of an interaction between CGRP and the immune system, which was summarized previously [1, 2]. Briefly, CGRP had an inhibitory effect on several aspects of the immune system, with reduced expression of CD86 and antigen presentation in dendritic cells, increased IL-10 expression in macrophages and monocytes, increased IL-17, IL-21, and IL-23 in Th17 cells, and decreased cytolytic activity in natural killers cells [3, 4]. Furthermore, CGRP negatively regulated the production of several cytokines (IL22, IFNγ, TNF⍺, IL23), shifting CD4 differentiation from Th1 and Th17 pathways [3, 4]. A speculative hypothesis for the observed cases was that inhibition of CGRP therefore promoted a Th1 phenotype and altered cytokine expression.

While there is pre-clinical evidence of an interaction between CGRP and the immune system, and clinical reports of inflammatory complications temporally related to commencement of therapy [2], the broader clinical significance remains unknown. Whether CGRP mAbs impact cytokine expression sub-clinically in an unselected migraine cohort is of relevance to determining the safety of these medications.

## Methodology

### Study objectives

The primary objective of the study was to determine if the initiation of CGRP monoclonal antibody therapy was associated with a change in cytokine profile in a non-selected population with chronic migraine. The secondary objective was to determine if cytokine levels were elevated in chronic migraine compared to healthy controls.

### Design

A prospective single-center observational analytic study with a case–control design. The study population included patients with chronic migraine according to the International Classification of Headache Disorders, third edition (ICHD-3) [5] who were commenced on a CGRP mAb according to local regulatory guidelines, which at the time of study recruitment allowed patients with chronic migraine, who had previously trialed three preventative medications and addressed medication overuse headache to be commenced on CGRP mAb [6], and excluded patients with co-morbid health conditions that may impact study results (e.g. auto-immune disorders), had previous exposure to a CGRP mAb, exposure to immuno-modulatory treatment, or who were not expected to be able to complete the study activities. Age and sex-matched healthy controls were recruited.

Participants’ diagnosis was confirmed by a headache sub-specialist as well as confirmation of demographic and clinical data. Participants donated blood prior to initiation of CGRP mAb and following three months of therapy. Healthy controls donated blood at a single timepoint.

### Evaluation

All participants provided informed consent prior to study activities. Participants were screened for intercurrent illness and donated plasma prior to commencement of CGRP mAb and after three months of continuous therapy. Participant samples were processed within two hours of collection. Samples underwent centrifugation and were stored in the Alfred Neurosciences Biobank (− 80 °C) until ready for analysis. Clinical evaluation of the efficacy of CGRP mAb was determined by the treating clinician following three months of therapy.

Sample analysis was undertaken on a Simoa^TM^ CorPlex human cytokine 10-plex (CPX) with ten targets: interferon gamma (IFNγ), interleukin (IL)-1β, IL-4, IL-5, IL-6, IL-8, IL-10, IL-12p70, IL-22, and tumor necrosis factor alpha (TNF⍺). The candidate cytokines targeted were chosen on the basis of the pre-clinical data on possible interactions with CGRP and the assays available [2]. Simoa CorPLex analysis was chosen for the low duplicate coefficient of variation (CV), lower limit of detection and quantification, sensitivity, and dynamic range (Quanterix, USA) [7].

### Statistical assessment

Statistical analysis was performed using SPSS v29.0. Population characteristics were summarized with descriptive statistics. For the study cohort, paired samples at baseline and following three months of therapy were assessed with a one-way repeated measures MANOVA, with post-hoc univariate analysis for each cytokine studied. Independent results—cytokine levels in the migraine population at baseline were compared to healthy controls and assessed by Mann–Whitney *U* test. Test results were considered significant when *p* < 0.05. This study received institutional review board approval (HREC 157/19).

## Results

A total of 22 cases and 10 controls completed the study. The mean age of cases was 50.6 (SD 12.3) and 86.4% of the population were female, consistent with the demographics of the disease. The mean age of patients with chronic migraine was 40.5 (SD 12.3), and the controls were 43.1 (SD 14.5). Cases and controls were well matched for age and sex. Population characteristics are available in Table 1. All patients had chronic migraine at baseline; 19/22 were commenced on galcanezumab and 3/22 on fremanezumab, reflecting the local drug availability at the time of recruitment. The mean duplicate coefficient of variation (CV) for the Simoa CorPlex cytokine panel was 3.9% for IFNγ, 4.3% for Il-1β, 7.0% for IL-4, 3.7% for IL-5, 3.6% for IL-6, 9.0% for IL-8, 4.7% for IL-10, 4.4% for IL-12p70, 6.1% for IL-22, and 4.8% for TNF⍺.

**Table 1:** Population characteristics

	Cases, *N* = 22	Controls, *N* = 10	
Mean age (SD)	40.5 (12.3)	43.1(14.5)	*p* = 0.683
Female (%)	19 (86.4)	8 (80)	*p* = 0.624
Median MHD at baseline (SD)	28 (9)	–	
Median MHD at follow-up (SD)	8 (18)	–	
Median previous preventers (IQR)	4 (2)	–	
CGRP mAb commenced *N* (%)			
Fremanezumab	3 (13.6)	–	
Galcanezumab	19 (86.4)	–	

*CGRP* mAb calcitonin gene-related peptide monoclonal antibody, *IQR* inter-quartile range, *MHD* monthly headache days, *SD* standard deviation

**Fig. 1:**
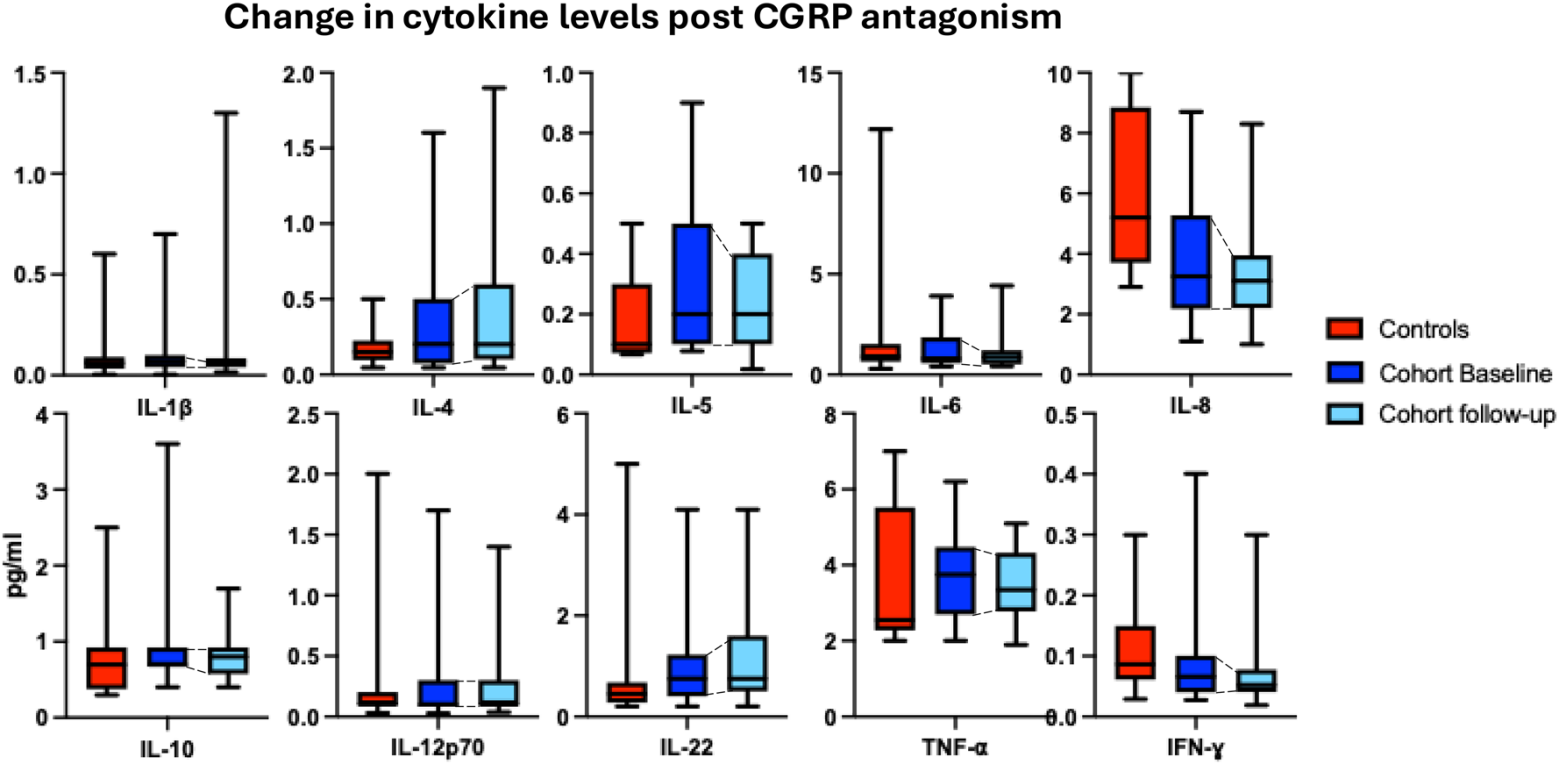
Change in cytokine level post CGRP antagonism

The primary objective of the study was to determine if initiation of CGRP mAb was associated with a change in plasma cytokine profile. Over a 3-month period of follow-up, administration of CGRP mAb was not associated with a change in plasma cytokine levels (Wilks’ Lambda 0.528, *p* = 0.448). On post-hoc analysis, there was a minor but significant reduction in IL-5 levels (*z* = − 2.321, *p* = 0.020), with median IL-5 levels reducing from 0.21 pg/ml to 0.19 pg/ml. There was no statistically significant change in IFNɣ, IL-10, IL-12p70, IL-1β, IL-22, IL-4, IL-6, IL-8, or TNF⍺ levels (Fig. 1).

The study had the secondary objective of comparing the cytokine profile of patients with chronic migraine to age and sex-matched controls. IL-8 levels were lower in the migraine population compared to healthy controls (*z* = − 2.602, *p* = 0.009), with a median level of 3.2 pg/ml in cases and 6.1 pg/ml in controls. There was no difference between the study groups for the remainder of the studied cytokines. Median cytokine levels are summarized in the supplemental material, Table 1.

## Discussion

Plasma cytokine levels have been studied previously in primary headache disorders, with higher levels of pro-inflammatory cytokines IL-6, IL-8, TNF-⍺, and IL-1β reported in migraine compared to healthy controls [8]. Our study is the first project to evaluate the cytokine profile of patients following exposure and treatment with CGRP monoclonal antibodies. There is no alteration of plasma cytokine profile following administration of CGRP mAb in this unselected patient cohort. A significant reduction in IL-5 was considered irrelevant due to the very small actual difference observed over the treatment course. There was no shift in Th1 cytokines.

IL-5 is secreted by Th2 cells, mast cells, eosinophils, and group 2 innate lymphoid cells (ILC2) [9]. IL-5 is a potent mediator of eosinophil maturation and has a role in the pathogenesis of asthma [9]. The cause of our observation of reduced IL-5 levels is not immediately apparent. While a small reduction in IL-5 was observed with treatment, there was not a significant variation between cases and healthy controls at baseline. Pre-clinically, CGRP limits ILC2 response, one of the mediators of IL-5 secretion [10, 11]. There are limited clinical studies evaluating IL-5 in patients with chronic migraine [8, 12]. In one study in 1998, Munno and colleagues found increased IL-5 levels in patients with migraine compared to healthy controls; however, this was not replicated in other studies [13, 14], including ours. Further longitudinal study is required to determine if the small change in plasma IL-5 level correlates with headache frequency, initiation of CGRP mAb, or whether this result represents a type I error.

Our second and probably more significant finding was that the patient cohort had substantially lower IL-8 levels compared to our healthy controls. While other studies have also found lower or no change in IL-8 levels in a migraine cohort [15, 16], these findings are in contrast to a 2023 meta-analysis of cytokine levels in headache disorders [8]. The meta-analysis found that IL-8 levels are higher in migraine than healthy controls, with a standardized mean difference of 1.56-fold (95% CI 0.03–3.09, *p* = 0.04). Given the putative role of inflammation in migraine chronification, our finding requires replication.

This study has several limitations that impact on the generalizability of our findings. Firstly, we did not include patients with pre-existing inflammatory conditions, and therefore the impact of CGRP monoclonal antibodies on patients with a dysregulated immune system cannot be surmised from this study, nor the impact of CGRP mAbs that target the receptor such as erenumab given local availability. Secondly, the size of the study did not allow for sub-group analysis, and a focus on mean levels might not reflect cohort heterogeneity. The impact of lifestyle change, non-pharmacological intervention, or use of non-steroidal anti-inflammatory as an acute therapy was not controlled for, which could also impact cytokine expression [17, 18].

## Conclusion

We found no evidence that treatment with CGRP monoclonal antibodies is associated with a significant alteration in plasma cytokine expression or shift to a Th1 phenotype in a cohort of patients with chronic migraine. Further study is required to determine the interaction of headache frequency and CGRP mAb therapy with IL-5 levels and in participants with pre-existing inflammatory conditions.

## Supplementary Information

Below is the link to the electronic supplementary material.

Supplementary file1 (DOCX 16 KB)

## References

[1] Ray JC, Kapoor M, Stark RJ et al (2021) Calcitonin gene related peptide in migraine: current therapeutics, future implications and potential off-target effects. J Neurol Neurosurg Psychiatry. 10.1136/jnnp-2020-324674 33495299 10.1136/jnnp-2020-324674

[2] Ray JC, Allen P, Bacsi A et al (2021) Inflammatory complications of CGRP monoclonal antibodies: a case series. J Headache Pain 22(1):121. 10.1186/s10194-021-01330-7 34625019 10.1186/s10194-021-01330-7 PMC8501661

[3] Assas BM, Pennock JI, Miyan JA (2014) Calcitonin gene-related peptide is a key neurotransmitter in the neuro-immune axis. Front Neurosci 8:23 24592205 10.3389/fnins.2014.00023 PMC3924554

[4] Sohn I, Sheykhzade M, Edvinsson L et al (2020) The effects of CGRP in vascular tissue - classical vasodilation, shadowed effects and systemic dilemmas. Eur J Pharmacol 881:173205 32442540 10.1016/j.ejphar.2020.173205

[5] The International Classification of Headache Disorders 3rd edition [Internet]. International Headache Society. 2019. Available from: https://ichd-3.org/. Cited 12 Aug 2019.

[6] PBS [Internet]. The Pharmaceutical Benefits Scheme. 2022. Available from: https://www.pbs.gov.au/pbs/home. Cited 15 Oct 2022

[7] Simoa^TM^ CorPlex^TM^ Human Cytokine Panel 1 SP-X® Data Sheet [Internet]. Quanterix. 2020. Available from: https://www.quanterix.com/wp-content/uploads/2020/12/CorPlex-Human-Cytokine-10-Plex-Panel-1-Data-Sheet.pdf. Cited 13 Jun 2024

[8] Musubire AK, Cheema S, Ray JC et al (2023) Cytokines in primary headache disorders: a systematic review and meta-analysis. J Headache Pain 24(1):36. 10.1186/s10194-023-01572-7 37016284 10.1186/s10194-023-01572-7 PMC10071234

[9] Matucci A, Maggi E, Vultaggio A (2019) Eosinophils, the IL-5/IL-5Rα axis, and the biologic effects of benralizumab in severe asthma. Respir Med 160:105819 31734469 10.1016/j.rmed.2019.105819

[10] Nagashima H, Mahlakõiv T, Shih H-Y et al (2019) Neuropeptide CGRP limits group 2 innate lymphoid cell responses and constrains type 2 inflammation. Immunity 51(4):682-695.e6 31353223 10.1016/j.immuni.2019.06.009 PMC6801073

[11] Wallrapp A, Burkett PR, Riesenfeld SJ et al (2019) Calcitonin gene-related peptide negatively regulates alarmin-driven type 2 innate lymphoid cell responses. Immunity 51(4):709-723.e6 31604686 10.1016/j.immuni.2019.09.005 PMC7076585

[12] Thuraiaiyah J, Erritzøe-Jervild M, Al-Khazali HM et al (2022) The role of cytokines in migraine: a systematic review. Cephalalgia 42(14):1565–1588. 10.1177/03331024221118924 35962530 10.1177/03331024221118924

[13] Munno I, Centonze V, Marinaro M et al (1998) Cytokines and migraine: increase of IL-5 and IL-4 plasma levels. Headache 38(6):465–467. 10.1046/j.1526-4610.1998.3806465.x 9664752 10.1046/j.1526-4610.1998.3806465.x

[14] Munno I, Marinaro M, Bassi A et al (2001) Immunological aspects in migraine: increase of IL-10 plasma levels during attack. Headache 41(8):764–767. 10.1046/j.1526-4610.2001.01140.x 11576199 10.1046/j.1526-4610.2001.01140.x

[15] Martami F, Razeghi Jahromi S, Togha M et al (2018) The serum level of inflammatory markers in chronic and episodic migraine: a case-control study. Neurol Sci 39(10):1741–1749. 10.1007/s10072-018-3493-0 30009333 10.1007/s10072-018-3493-0

[16] Cowan RP, Gross NB, Sweeney MD et al (2021) Evidence that blood–CSF barrier transport, but not inflammatory biomarkers, change in migraine, while CSF sVCAM1 associates with migraine frequency and CSF fibrinogen. Headache J Head Face Pain. 61(3):536–45. 10.1111/head.14088 10.1111/head.14088 PMC8023403 33724462

[17] Housby JN, Cahill CM, Chu B et al (1999) Non-steroidal anti-inflammatory drugs inhibit the expression of cytokines and induce HSP70 in human monocytes. Cytokine 11(5):347–358 10328874 10.1006/cyto.1998.0437

[18] D’Esposito V, Di Tolla MF, Lecce M et al (2022) Lifestyle and dietary habits affect plasma levels of specific cytokines in healthy subjects. Front Nutr. 10.3389/fnut.2022.913176/full 35811952 10.3389/fnut.2022.913176 PMC9270017

