# Supplementary material for: The impact of CGRP monoclonal antibodies on cytokine expression in chronic migraine: a cohort study
Source: J Neurol. 2025 Sep 24;272(10):649. doi: 10.1007/s00415-025-13400-w (PMC12460511; doi:10.1007/s00415-025-13400-w)
Supplement: Supplementary file 1 — Supplementary file1 (DOCX 16 KB) [file 415_2025_13400_MOESM1_ESM.docx]

**Supplemental material**

|  | Baseline  N=22 | Follow-up  N=22 | Paired samples* | Controls  N=10 | Cases vs Controls^ |
| --- | --- | --- | --- | --- | --- |
| Median IFNɣ  (pg/ml) | 0.0657 (0.1028) | 0.0523 (0.037) | P=0.088 | 0.0933 (0.1099) | P=0.193 |
| Median IL-10  (pg/ml) | 0.7437 (0.6828) | 0.7769 (0.3564) | P=0.783 | 0.6786 (0.5862) | P=0.393 |
| Median IL-12p70  (pg/ml) | 0.1268 (0.2197) | 0.1302 (0.1532) | P=0.758 | 0.1348 (0.0704) | P=0.935 |
| Median IL-1β  (pg/ml) | 0.07 (0.0813) | 0.0671 (0.0446) | P=0.548 | 0.0605 (0.0596) | P=0.440 |
| Median IL-22  (pg/ml) | 0.7489 (0.8) | 0.7477 (1.0626) | P=0.485 | 0.4464 (0.3976) | P=0.080 |
| Median IL-4  (pg/ml) | 0.1779 (0.4028) | 0.2265 (0.4817) | P=0.140 | 0.1661 (0.3158) | P=0.640 |
| Median IL-5  (pg/ml) | 0.2055 (0.3588) | 0.1946 (0.2920) | P=0.020 | 0.1528 (0.3158) | P=0.309 |
| Median IL-6  (pg/ml) | 0.8168 (1.2838) | 0.8217 (0.6683) | P=0.527 | 0.8952 (0.8781) | P=0.837 |
| Median IL-8  (pg/ml) | 3.237 (3.1144) | 3.2446 (2.0005) | P=0.592 | 6.0996 (6.6481) | P=0.009 |
| Median TNF⍺  (pg/ml) | 3.7774 (1.7542) | 3.3316 (1.5015) | P=0.808 | 2.5549 (3.2412) | P=0.440 |

Table 1 Cytokine levels at baseline, follow-up and for healthy controls. IFNɣ; interferon-ɣ, IL; interleukin, TNF⍺; tumour necrosis factor-⍺. *P values for univariate paired-samples at baseline and follow-up in cases. ^P-values for cytokine levels in cases vs healthy controls.
